# A Good Work

**Published:** 1888-12

**Authors:** 


### A GOOD WORK.

The Young Woman's Christian Association of Boston, has established a school for young women, of practical Housekeeping, embracing all that pertains to the management of the household. We congratulate them upon a step so thoroughly Christian. The course covers every department of the complete home, including the kitchen, decoration and dress, thus calling into requisition a knowledge of many of the industrial arts.

This enterprise should meet with a generous support.

